# Lessons learned for preventing health disparities in future pandemics: the role of social vulnerabilities among children diagnosed with severe COVID-19 early in the pandemic

**DOI:** 10.3934/publichealth.2025009

**Published:** 2025-01-15

**Authors:** Kelly Graff, Ye Ji Choi, Lori Silveira, Christiana Smith, Lisa Abuogi, Lisa Ross DeCamp, Jane Jarjour, Chloe Friedman, Meredith A. Ware, Jill L Kaar

**Affiliations:** 1 Department of Pediatrics, Medical College of Wisconsin, Milwaukee, WI, USA; 2 University of Colorado School of Public Health, Aurora, CO, USA; 3 Department of Pediatrics, Anschutz Medical Campus, University of Colorado, Aurora, CO, USA; 4 Department of Pediatrics, Boston Children's Hospital, Boston, MA, USA; 5 Department of Pediatrics, David Geffen School of Medicine at UCLA, Los Angeles, CA, USA

**Keywords:** COVID-19, children, ethnic disparities, health disparities, obesity, social vulnerability, social determinants or health

## Abstract

**Background:**

Hispanic ethnicity is associated with an increased risk for severe disease in children with COVID-19. Identifying underlying contributors to this disparity can lead to improved health care utilization and prevention strategies.

**Methods:**

This is a retrospective cohort study of children 2–20 years of age with positive SARS-CoV-2 testing from March–October 2020. Univariable and multivariable logistic regression models were fitted to identify demographic, comorbid health conditions, and social vulnerabilities as predictors of severe COVID-19 (need for hospital admission or respiratory support).

**Results:**

We included 1572 children with COVID-19, of whom 45% identified as Hispanic. Compared to non-Hispanic children, patients who identified as Hispanic were more often obese (28% vs. 14%, p < 0.0001), preferred a non-English language (31% vs. 3%, p < 0.0001), and had Medicaid or no insurance (79% vs. 33%, p < 0.0001). In univariable analyses, children who identified as Hispanic were more likely to require hospital admission (OR 2.4, CI: 1.57–3.80) and respiratory support (OR 2.4, CI: 1.38–4.14). In multivariable analyses, hospital admission was associated with obesity (OR 1.9, CI: 1.15–3.08), non-English language (OR 2.4, CI: 1.35–4.23), and Medicaid insurance (OR 2.0, CI: 1.10–3.71), but ethnicity was not a significant predictor of severe disease.

**Conclusions and Relevance:**

The high rates of severe COVID-19 observed in Hispanic children early in the pandemic appeared to be secondary to underlying co-morbidities and social vulnerabilities that may have influenced access to care, such as language and insurance status. Pediatric providers and public health officials should tailor resource allocation to better target this underserved patient population.

## 1. Introduction

As of August 2023, over 15 million U.S. children had been diagnosed with coronavirus disease 2019 (COVID-19) and nearly 200,000 required hospital admission [1],[2]. Children in minority communities, particularly Hispanic and non-Hispanic Black children, were found to be disproportionately affected by higher rates of severe acute respiratory syndrome coronavirus-2 (SARS-CoV-2) acquisition and more severe COVID-19 early in the pandemic [3]–[13] and were less pronounced as the pandemic progressed [14]–[16]. A recent study from Colorado in May–July 2021 found that SARS-CoV-2 seroprevalence was highest among Hispanic children compared to other racial and ethnic groups, but they had lower confirmed case counts, suggesting reduced rates of testing among Hispanic children [17]. The underlying driver of these disparities remains unknown, though it has been hypothesized to be associated with a combination of comorbid health conditions, socioeconomic, and/or sociodemographic factors. To ensure public health efforts are positioned to address such disparities for similar infectious diseases, research needs to identify such drivers to improve future prevention strategies.

People who identify as Hispanic or Latino comprise 25% of the U.S. population and make up the largest minority group in Colorado [18],[19]. Preceding the COVID-19 pandemic, the health of Hispanic children in the U.S. was impacted by disparities [20]–[23]. For example, Hispanic children have higher rates of obesity than other racial/ethnic groups, and Hispanic children whose parents immigrated to the U.S. (i.e., 2nd generation) have the highest rates of obesity [20]–[23]. Obesity and other pre-existing health conditions have been identified as clear risk factors for severe COVID-19 in children, but whether ethnicity contributes additional risk needs further exploration [6],[24]–[26].

The Children and COVID-19 in Colorado (CCC) study has established one of the largest cohorts of pediatric COVID-19 patients in the U.S., leveraging Children's Hospital Colorado (CHCO)'s COVID-19 testing network. We previously described a disproportionate burden of COVID-19 infection and hospitalization in Hispanic children in Colorado [6]. In this study, we identify the underlying demographic, comorbid health conditions, and social vulnerabilities that may predict severe COVID-19 among children with Hispanic ethnicity.

## 2. Materials and methods

### 2.1. Study population and data collection

This is a retrospective cohort study of patients who tested positive for SARS-CoV-2 at CHCO from March 15 to October 31, 2020. This period was selected to capture the early phase of the COVID-19 pandemic, as prior studies have identified this period to be when disparities in outcomes between Hispanic and non-Hispanic populations were most pronounced [15]. Children's Hospital Colorado (CHCO) is the primary referral center for children in a seven-state region. The network provides comprehensive care, including emergency, inpatient, outpatient, and subspeciality care. The community surrounding the main campus includes 40% racial/ethnic minority, with the largest minority population identified as Hispanic, with nearly 20% of children living at or below poverty level [27]. Records were collected from all CHCO health system locations, including a 434-bed acute care hospital in Aurora, CO, a 111-bed acute care hospital in Colorado Springs, CO, and 13 additional network locations offering outpatient, specialty, and urgent care. All sites use a common electronic health record (EHR; Epic systems, Verona, WI). Demographic and clinical data were manually abstracted from the EHR and entered into standardized data collection forms developed in REDCap, hosted by the University of Colorado, Denver [28]. The study was approved by the Colorado Multiple Institutional Review Board (COMIRB protocol 20–0972) with a waiver of informed consent as secondary research.

### 2.2. Inclusion criteria and definitions of variables

**Figure 1.**
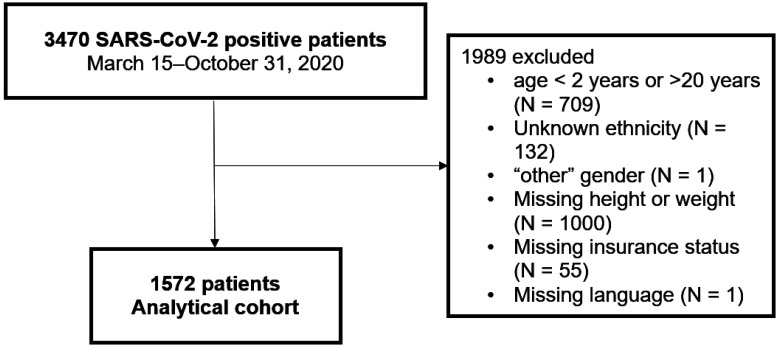
Inclusion and exclusion criteria.

Children were included if they tested positive for SARS-CoV-2 on a validated nucleic acid amplification test at any CHCO location during the study period [6]. All variables were abstracted as documented in the EHR. Age was reported at first positive COVID-19 detection. To ensure key study variables (e.g., obesity) could be compared across varying age groups of children and youth, children between the ages of 2 and 20 years were included. Age was further categorized as 2–5 years, 6–10 years, 11–15 years, and 16–20 years. Sex was categorized as male or female; other genders were excluded from analysis. Ethnicity was categorized as Hispanic or non-Hispanic, based on patient/parent self-report. Race was categorized as White, Black, more than one race, or other. This analysis focused on ethnicity instead of race, as there was no association between race and severe COVID-19 in our previous analysis [6]. Insurance type and preferred written language were used as proxies to reflect social vulnerabilities. Additional negative social determinants of health (SDoH) or needs (e.g., food insecurity) were not routinely collected across the study health system at time of data collection; Insurance type was categorized into either non-Medicaid (i.e., private insurance) or the combined category of Medicaid or uninsured (i.e., Health First, CHP+, charity care). Preferred written language was categorized as either English or non-English. Household size was calculated as the total number of people, including the patient, living in the home at the time of the positive SARS-CoV-2. Patients with unknown ethnicity, preferred language, or insurance status were excluded (Figure 1).

Body mass index (BMI) was calculated from the height and weight documented closest to the date of COVID-19 detection, ±6 months for patients between 24 months and 5 years old, and ±12 months for patients greater than 5 years old. BMI was categorized into percentiles based on the Centers for Disease Control and Prevention (CDC) growth charts and definition of underweight (<5th percentile), healthy weight (5–85th percentile), overweight (>85th percentile) and obese (BMI percentile >95th) [29],[30]. Patients missing height and/or weight data to calculate BMI were excluded. Specific comorbid conditions known to be related to obesity were obtained from the patients' problem list, including diabetes/pre-diabetes, sleep apnea, hyperlipidemia, hypertension, and nonalcoholic fatty liver disease (NAFLD) or nonalcoholic steatohepatitis (NASH). Severe cases of COVID-19 were defined as requiring hospital admission with one or more COVID-19-related symptoms (fever above 100.4F, chills, cough, congestion/runny nose, shortness of breath, etc.); a subgroup of patients was classified further as requiring any respiratory support (i.e., low flow or high flow nasal cannula, non-invasive positive pressure ventilation, intubation/ventilation, or ECMO) during COVID-19 related admission.

### 2.3. Statistical analysis

The statistical analysis for this paper was generated using SAS software, version 9.4_M7 (SAS Institute Inc., Cary, NC, USA). Descriptive statistics evaluated group differences between ethnicity and demographic, socioeconomic, and comorbid health conditions using Pearson Chi-Square test or Fisher's Exact test for categorical data and the T-tests or Wilcoxon tests for continuous data. Categorical variables were summarized by the number of patients and frequencies as percentages; continuous variables were summarized by median and interquartile range based on distribution. To address our main research question and examine predictors of severe COVID-19, a multivariable logistic regression model was fitted. All demographic, comorbid health conditions, and social vulnerabilities that were significant predictors of severe COVID-19 with p < 0.10 in univariable models were then entered into multivariable logistic model. Comorbidities with less than 30 observed cases were not explored further in multivariable analyses due to concern for underpowered tests. The p-value ≤ 0.05 was considered statistically significant in multivariable models.

### 2.4. Ethics approval

This study was approved by the Colorado Multiple Institutional Review Board with a waiver of informed consent as secondary research.

## 3. Results

Between March 15 and October 31, 2020, a total of 3470 children with SARS-CoV-2 were identified at CHCO (Figure 1). After excluding participants based on age (n = 709), gender (n = 1), or missing the variables of ethnicity (n = 132), height or weight (n = 1000), language (n = 1), or insurance status (n = 55), a total of 1572 children remained, with a median age of 11.2 years (48% female, 45% Hispanic or Latino) (Table 1). The median BMI percentile for age and sex for Hispanic children was significantly higher compared to non-Hispanic children (70% vs. 59%; p < 0.0001). Further, Hispanic children were more likely to have Medicaid insurance or be uninsured (79% vs. 33%; p < 0.0001); Hispanic families were more likely to prefer a non-English language (31% vs. 3%; p < 0.0001); and Hispanic families had a larger median household size (4.5 vs. 4.1 persons, p < 0.0001). Hispanic children diagnosed with COVID-19 had a higher median number of obesity and obesity-related comorbid conditions compared to non-Hispanic children (1.2 vs. 0.7; p < 0.0001) (Table 2). Twenty percent of all children diagnosed with COVID-19 had a diagnosis of obesity, with significant differences in the proportions of children with obesity between Hispanic and non-Hispanic patients (28% vs. 14%; p < 0.0001). Several obesity-related comorbid health conditions such as hyperlipidemia, NAFLD or NASH, and sleep apnea were significantly more common in Hispanic versus the non-Hispanic children. However, hypertension and diabetes were not significantly different between the two groups.

Of the 1572 children diagnosed with COVID-19, 83 (5%) children were admitted to the hospital with COVID-19 related symptoms. Of those admitted (n = 83), 54 (65%) required additional respiratory support. Children who identified as Hispanic were more often admitted (7% vs. 4%; p < 0.0001) and more often required respiratory support (5% vs. 2%; p = 0.002) compared to non-Hispanic children.

The univariable model identified age, ethnicity, sleep apnea, obesity, preferred language, and insurance to be significantly associated with both outcomes measuring severe COVID-19: hospital admission (Table 3) and hospital admission plus need for respiratory support (Table 4).

In the multivariable analysis, variables that remained significant predictors of hospital admission included older age [Odds Ratio (OR) 2.3, 95% CI: 1.3–4.2], sleep apnea (OR 4.2, 95% CI: 2.2–8.1), or obesity (OR 1.9, 95% CI: 1.2–3.1) (Table 3). Further, patients were more than twice as likely to require admission if they reported a preferred language other than English (OR 2.4, 95% CI: 1.4–4.2) or had Medicaid or no insurance (OR 2.0, 95% CI: 1.1–3.7). Similarly, the multivariable analysis found that significant predictors of need for respiratory support included older age (OR 2.3, 95% CI: 1.1–4.8) or having a diagnosis of sleep apnea (OR 4.2, 95% CI: 1.9–9.5) or obesity (OR 2.6, 95% CI: 1.4–4.6) (Table 4). The odds of requiring respiratory support were also significantly increased among children with a preferred language other than English (OR 4.1, 95% CI: 2.1–8.2) or who had Medicaid or no insurance (OR 2.9, 95% CI: 1.3–6.5). However, ethnicity was no longer a significant predictor of severe COVID-19 in either multivariable analysis.

**Table 1.** Demographic characteristics of children by ethnicity.

	**All ** **(N = 1572)**	**Hispanic or Latino ** **(n = 702)**	**Non-Hispanic or Latino ** **(n = 870)**	**p-value**
**Median (IQR) Age, years**	11.2 ± 5.3	11.0 ± 5.1	11.3 ± 5.4	0.38
**Age Group, years**				**0.04**
2–5	357 (23%)	149 (21%)	208 (24%)	
6–10	375 (24%)	187 (27%)	188 (22%)	
11–15	446 (28%)	206 (29%)	240 (28%)	
16–20	394 (25%)	160 (23%)	234 (27%)	
**Race**				**<0.001**
White	934 (61%)	286 (42%)	648 (77%)	
African				
American/Black	79 (5%)	4 (1%)	75 (9%)	
More than one race	97 (6%)	53 (8%)	44 (5%)	
Other	381 (26%)	316 (46%)	65 (8%)	
Not reported	33 (2%)	21 (3%)	12 (1%)	
**Sex**				0.40
Male	810 (52%)	353 (50%)	457 (53%)	
**Weight Status**				**<0.001**
Underweight	80 (5%)	23 (3%)	57 (7%)	
Healthy Weight	932 (59%)	360 (51%)	572 (66%)	
Overweight	238 (15%)	119 (17%)	119 (14%)	
Obese	322 (21%)	200 (28%)	122 (14%)	
**Insurance**				**<0.0001**
Medicaid or uninsured	848 (54%)	558 (79%)	290 (33%)	
Medicaid	830 (53%)	546 (78%)	284 (33%)	
Uninsured	18 (1%)	12 (2%)	6 (1%)	
Private	724 (46%)	144 (21%)	580 (67%)	
**Preferred Language**				**<0.0001**
Non-English	245 (16%)	219 (31%)	26 (3%)	
**Median Household Size**	4.3 ± 1.5	4.5 ± 1.6	4.1 ± 1.3	**<0.0001**

**Table 2.** Obesity-related medical conditions of children by ethnicity.

	**All ** **(N = 1572)**	**Hispanic or Latino** **(n = 702)**	**Non-Hispanic or Latino ** **(n = 870)**	**p-value**
**Comorbidities^a^**				
At least one	601 (64%)	331 (67%)	270 (61%)	0.05
Median (IQR) number	0.9 ± 1.8	1.2 ± 2.0	0.7 ± 1.6	<0.0001
**Comorbid Condition**				
Hypertension	8 (1%)	6 (1%)	2 (0%)	0.15
Hyperlipidemia	12 (1%)	10 (1%)	2 (0%)	0.02
NAFLD/NASH^b^	11 (1%)	11 (2%)	0 (0%)	<0.001
Sleep Apnea	80 (5%)	48 (7%)	32 (4%)	<0.001
Diabetes/pre-diabetes	21 (1%)	10 (1%)	11 (1%)	0.96

Note: ^a^Obesity is not included in this table of obesity-related comorbidities; ^b^NAFLD = nonalcoholic fatty liver disease; NASH = nonalcoholic steatohepatitis.

**Table 3.** Demographic, health conditions, and social vulnerabilities associated with need for hospital admission.

	**Univariable OR ** **(95% CI)**	**p-value**	**Multivariable OR ** **(95% CI)**	**p-value**
**Demographics**				
Age (years)				
2–5	0.8 (0.4, 1.7)	0.59	0.91 (0.4, 1.9)	0.79
6–10	0.7 (0.3, 1.4)	0.28	0.7 (0.3, 1.5)	0.37
11–15	Ref		Ref	
16–20	2.0 (1.2, 3.6)	**0.01**	2.5 (1.4, 4.5)	**0.01**
Ethnicity (Hispanic or Latino)	2.0 (1.2, 3.1)	**0.01**	0.9 (0.5, 1.6)	0.77
Sex (Female)	1.2 (0.8, 1.9)	0.33		
**Health Condition^a^**				
Sleep apnea	5.3 (2.9, 9.7)	**<0.0001**	5.3 (2.8, 10.0)	**<0.0001**
Obese	2.6 (1.6, 4.1)	**<0.0001**	2.0 (1.2, 3.2)	**0.01**
**Social Vulnerabilities**				
Preferred Language (not English)	3.0 (1.9, 4.8)	**<0.0001**	2.3 (1.3, 4.1)	**<0.01**
Insurance (Medicaid or uninsured)	3.0 (1.8, 5.1)	**<0.0001**	2.1 (1.1, 3.8)	**0.02**
Household size	1.1 (0.9, 1.3)	0.46		

Note: ^a^Not included in analysis due to small sample size: Hypertension, Hyperlipidemia, NAFLD/NASH, Diabetes/pre-diabetes.

**Table 4.** Demographic, health conditions, and social vulnerabilities associated with need for respiratory support.

	**Univariable OR ** **(95% CI)**	**p-value**	**Multivariable OR ** **(95% CI)**	**p-value**
**Demographics**				
Age (years)				
2–5	0.6 (0.3, 1.6)	0.30	0.7 (0.3, 1.8)	0.46
6–10	0.9 (0.4, 1.9)	0.69	0.9 (0.4, 2.2)	0.89
11–15	Ref		Ref	
16–20	1.9 (1.0, 3.8)	**0.06**	2.4 (1.2, 5.0)	**0.02**
Ethnicity (Hispanic or Latino)	2.0 (1.1, 3.5)	**0.02**	0.6 (0.3, 1.2)	0.16
Sex (Female)	1.2 (0.7, 2.1)	0.48		
**Health condition**				
Sleep apnea	4.7 (2.3, 9.7)	**<0.0001**	4.7 (2.2, 10.3)	**0.0001**
Obese	3.5 (2.1, 6.1)	**<0.0001**	2.6 (1.4, 4.7)	**0.01**
**Social Vulnerabilities**				
Preferred Language (not English)	4.7 (2.7, 8.2)	**<0.0001**	4.0 (2.0, 8.0)	**<0.0001**
Insurance (Medicaid or Uninsured)	4.5 (2.2, 9.2)	**<0.0001**	2.9 (1.4, 4.7)	**0.01**
Household size	1.1 (0.9, 1.4)	0.26		

## 4. Discussion

This study demonstrates that although we observed an increase in the prevalence of severe COVID-19 among Hispanic children early in the COVID-19 pandemic, ethnicity was not the driving factor, but rather the relationship was confounded by several social vulnerabilities and pre-existing health conditions that were more common in Hispanic children. Specifically, children with comorbidities such as obesity and sleep apnea, and indicators of an immigrant family (family preference for non-English language) or lower socioeconomic status (public insurance or no insurance), were more likely to experience severe COVID-19.

Obesity and obesity-related comorbidities are more common in Hispanic patients as demonstrated in previous literature [20]–[23] and as confirmed by our analysis. In addition to obesity, our study demonstrates that obesity-related comorbidities, such as sleep apnea, are independently associated with more severe COVID-19 outcomes. Interestingly, diabetes/pre-diabetes was not associated with more severe disease. However, the patients with diabetes in our cohort were diagnosed with type 1 or type 2 or pre-diabetes, and therefore are not all expected to be linked with obesity. This potentially explains why we did not see diabetes as a predictor for COVID-19 severity.

Obesity has also been shown to be associated with low socioeconomic status (SES) and among US-born children with Latino immigrant parents [20],[21]. SDoH such as public insurance status and preferred non-English language were independently associated with more severe COVID-19 outcomes. These factors may be representative of an immigrant or low-income population with lower access to health care, which may contribute to the disparity in COVID-19 outcomes [31],[32]. Recent studies support this by demonstrating a disproportionate decrease in emergency department utilization among minorities during the pandemic and decreased acute care utilization in those in low Childhood Opportunity Index (COI) neighborhoods [33],[34]. The authors of the study suggest that COVID-19 mitigation efforts during the pandemic may have led to a further decrease in healthcare access for these already underserved populations, for example due to a decrease in transportation access [34],[35]. Further, a survey of parents on hesitancy to seek healthcare demonstrated that parents of children with social vulnerabilities, such as those living in low COI neighborhoods, non-English speaking, non-white race, and those with public payor sources, were more hesitant to seek care at the start of the pandemic [36].

Our previous analysis was unable to disentangle obesity and social vulnerabilities from ethnicity. This multivariable model demonstrates that obesity, obesity-related conditions, and social vulnerabilities are stronger predictors of severe COVID-19 than ethnicity itself. Although the univariable analyses demonstrate that each individual factor is a predictor of severe disease, in the multivariable analyses, obesity, sleep apnea, and social vulnerabilities are independently associated. Further, these factors are consistent predictors for severe disease in both models, need for hospitalization and need for respiratory support.

Importantly, this cohort pre-dated the approval of COVID-19 vaccines for children, and therefore, vaccination status did not affect severity outcomes. The COVID-19 vaccine has since become widely available and many public health efforts have primarily focused on targeting racial/ethnic minorities in vaccination campaigns. Our study suggests the need to take efforts further and focus specifically on immigrant families with low acculturation, specifically those in non-English speaking communities and low-income neighborhoods. Targeting efforts towards underserved populations via community-based approaches may provide the greatest benefit. Further, as Hispanic children were disproportionately affected early in the pandemic, future efforts need to be focused on targeting interventions for the most vulnerable populations early on in future pandemics and respiratory viral seasons.

Our study has potential limitations. First, this study was conducted in a single healthcare system in Colorado, and therefore results may not be generalizable to other settings. This was a retrospective study with several missing data for some variables that led to exclusion of a large number of patients. In particular, 45% of the patients included in the analysis were Hispanic, whereas only 35% of patients excluded for missing height and weight were Hispanic; this may have led to unanticipated biases. However, when comparing the children that were excluded due to missing height or weight, differences in patient characteristics between Hispanic and non-Hispanic patients were similar to the analytic cohort (Supplemental Table 1). As the data was limited to what was available in the EHR, additional negative social determinants of health and other sociodemographic factors related to health outcomes could not be explored, including education, family income, and social vulnerability index. Additionally, this cohort only included children with positive SARS-CoV-2 PCR, and therefore did not assess if there were disparities in those obtaining SARS-CoV-2 testing. However, a cross-sectional serosurvey conducted in Colorado found that seroprevalence was higher among Hispanic children compared to non-Hispanic children despite lower COVID-19 case ascertainment in Hispanic children based on PCR testing [17]. Despite this, we identified significant factors associated with Hispanic/Latino ethnicity that predicted more severe COVID-19 outcomes. Further, this analysis focused on obesity and obesity-related comorbidities only. It is known that other comorbidities predict severe disease in COVID-19, as demonstrated in our previous analysis [6]. The current analysis focuses on obesity-related comorbidities only, in an effort to disentangle this association with ethnicity. Finally, changing recommendations and selection bias on testing and treatment throughout the pandemic may have also impacted outcomes; however, our cohort was restricted to the first 8 months of the pandemic before vaccine introduction and new variants arose. Therefore, we do not anticipate that this created significant bias. Despite these limitations, our study is one of the first to attempt to disentangle the many confounding factors associated with increased rates of severe COVID-19 outcomes that have been observed among Hispanic children in the U.S.

## 5. Conclusions

Clear health disparities exist among children who identify as Hispanic or Latino, and those disparities were amplified during the early COVID-19 pandemic. These disparities appear to be driven primarily by increased rates of obesity and obesity-related health conditions, among children in immigrant families, and/or with low SES. Public health efforts should target these vulnerable populations during this pandemic, future pandemics, and respiratory seasons.

## Use of AI tools declaration

The authors declare they have not used Artificial Intelligence (AI) tools in the creation of this article.


