# Supplementary material for: Lessons learned for preventing health disparities in future pandemics: the role of social vulnerabilities among children diagnosed with severe COVID-19 early in the pandemic
Source: AIMS Public Health. 2025 Jan 15;12(1):124–36. doi: 10.3934/publichealth.2025009 (PMC11999811; doi:10.3934/publichealth.2025009)
Supplement: Supplementary file 1 [file publichealth-12-01-009-s001.pdf]

Public Health

AIMS Public Health, 12(1): 124–136.
DOI: 10.3934/publichealth.2025009
Received: 03 July 2024
Revised: 09 September 2024
Accepted: 12 November 2024
Published: 15 January 2025

https://www.aimspress.com/journal/aimsph

Research article

Lessons learned for preventing health disparities in future pandemics: the role of social vulnerabilities among children diagnosed with severe COVID-19 early in the pandemic

Kelly Graff[1,*], Ye Ji Choi[2], Lori Silveira[3], Christiana Smith[3], Lisa Abuogi[3], Lisa Ross DeCamp[3], Jane Jarjour[4], Chloe Friedman[2], Meredith A. Ware[5] and Jill L Kaar[3]

1 Department of Pediatrics, Medical College of Wisconsin, Milwaukee, WI, USA
2 University of Colorado School of Public Health, Aurora, CO, USA
3 Department of Pediatrics, Anschutz Medical Campus, University of Colorado, Aurora, CO, USA
4 Department of Pediatrics, Boston Children's Hospital, Boston, MA, USA
5 Department of Pediatrics, David Geffen School of Medicine at UCLA, Los Angeles, CA, USA

* **Correspondence:** Email: kegraff@mcw.edu; Tel: +4142664123.

Supplementary

Supplemental Table 1. Characteristics of Children by Ethnicity who were excluded from the analysis for missing height or weight.

	**All (N = 741)***	**Hispanic or Latino (n=256)**	**Non-Hispanic or Latino (n=485)**	**p-value**
**Median (IQR) Age, years**	11.2 ± 5.3	10.6 ± 5.1	11.2 ± 5.3	0.12
**Age Group, years**				0.10
2–5	176 (24%)	60 (23%)	116 (24%)	
6–10	182 (25%)	72 (28%)	110 (23%)	
11–15	202 (27%)	74 (29%)	128 (26%)	
16–20	181 (24%)	50 (20%)	131 (27%)	
**Race**				**<0.001**
White	504 (69%)	104 (41%)	400 (85%)	
African American/Black	21 (3%)	0 (0%)	21 (4%)	
More than one race	38 (5%)	23 (9%)	15 (3%)	
Other	139 (19%)	116 (46%)	23 (5%)	
Not reported	24 (3%)	10 (4%)	14 (3%)	
**Sex**				0.27
Male	394 (53%)	129 (50%)	265 (55%)	
**Insurance**				**<0.0001**
Medicaid or uninsured	282 (38%)	196 (77%)	86 (18%)	
Medicaid	273 (37%)	190 (74%)	83 (17%)	
Uninsured	9 (1%)	6 (3%)	3 (1%)	
Private	440 (59%)	54 (21%)	386 (80%)	
**Preferred Language**				**<0.0001**
Non-English	80 (11%)	74 (29%)	0 (0%)	

Note: *Although, there were 1000 children excluded for missing height or weight, this number excludes 259 children who also had missing ethnicity data.

AIMS Press

© 2025 the Author(s), licensee AIMS Press. This is an open access article distributed under the terms of the Creative Commons Attribution License (https://creativecommons.org/licenses/by/4.0)
